# Investigation of Digital Technology Use in the Transition to Parenting: Qualitative Study

**DOI:** 10.2196/25388

**Published:** 2021-02-17

**Authors:** Lorie Donelle, Jodi Hall, Bradley Hiebert, Kimberley Jackson, Ewelina Stoyanovich, Jessica LaChance, Danica Facca

**Affiliations:** 1 Arthur Labatt Family School of Nursing Faculty of Health Science Western University London, ON Canada; 2 Faculty of Health, Community Studies and Public Safety Fanshawe College London, ON Canada; 3 Faculties of Health Science and Information and Media Studies Western University London, ON Canada

**Keywords:** parenting, digital health, technology, health literacy, information seeking

## Abstract

**Background:**

The transition to parenting—that is, the journey from preconception through pregnancy and postpartum periods—is one of the most emotionally charged and information-intense times for individuals and families. While there is a developing body of literature on the use and impact of digital technology on the information behaviors of children, adolescents, and young adults, personal use of digital technology during the transition to parenting and in support of infants to 2 years of age is relatively understudied.

**Objective:**

The purpose of this study was to enhance our understanding of the ways digital technologies contribute to the experience of the transition to parenting, particularly the role these technologies play in organizing and structuring emerging pregnancy and early parenting practices.

**Methods:**

A qualitative descriptive study was conducted to understand new parents’ experiences with and uses of digital technology during 4 stages—prenatal, pregnancy, labor, and postpartum—of their transition to becoming a new parent. A purposive sampling strategy was implemented using snowball sampling techniques to recruit participants who had become a parent within the previous 24 months. Focus groups and follow-up interviews were conducted using semistructured interview guides that inquired about parents’ type and use of technologies for self and family health. Transcribed audio recordings were thematically analyzed.

**Results:**

A total of 10 focus groups and 3 individual interviews were completed with 26 participants. While recruitment efforts targeted parents of all genders and sexual orientations, all participants identified as heterosexual women. Participants reported prolific use of digital technologies to direct fertility (eg, ovulation timing), for information seeking regarding development of their fetus, to prepare for labor and delivery, and in searching for a sense of community during postpartum. Participants expressed their need for these technologies to assist them in the day-to-day demands of preparing for and undertaking parenting, yet expressed concerns about their personal patterns of use and the potential negative impacts of their use. The 3 themes generated from the data included: “Is this normal; is this happening to you?!”, “Am I having a heart attack; what is this?”, and “Anyone can put anything on Wikipedia”: Managing the Negative Impacts of Digital Information.

**Conclusions:**

Digital technologies were used by mothers to track menstrual cycles during preconception; monitor, document, and announce a pregnancy during the prenatal stage; prepare for delivery during labor/birth stage; and to help babies sleep, document/announce their birth, and connect to parenting resources during the postpartum stage. Mothers used digital technologies to reassure themselves that their experiences were normal or to seek help when they were abnormal. Digital technologies provided mothers with convenient means to access health information from a range of sources, yet mothers were apprehensive about the credibility and trustworthiness of the information they retrieved. Further research should seek to understand how men and fathers use digital technologies during their transition to parenting. Additionally, further research should critically examine how constant access to information affects mothers’ perceived need to self-monitor and further understand the unintended health consequences of constant surveillance on new parents.

## Introduction

The transition to parenting—that is, the journey from preconception through pregnancy and postpartum periods—is one of the most emotionally charged and information-intense times for individuals and families [1-3]. During this time, resources that are often highly valued by transitioning parents include midwives, physicians, family and friends, and increasingly, internet-based resources [4]. Additionally, the use of digital technologies—broadly defined as devices such as computers, video cameras, and gaming systems; mobile devices such as phones and smartphones; and all applications (eg, internet) that are computer-dependent—constitutes the fastest growing information resource used by families as they negotiate the transition to parenting [5,6]. While there is a developing body of literature on the use and impact of digital technology on the information behaviors of children, adolescents, and young adults [7-10], personal use of digital technology during the transition to parenting and in support of infants to 2 years of age is relatively understudied.

With the rapid proliferation and uptake of digital technologies, it is crucial to understand which technologies are used by parents to make decisions impacting their health and that of their families and how their use contributes to the transition to parenting in both virtual and material spaces and at their intersections [11-13]. Recent information suggests that pregnant women and mothers of young children value information gathered from digital sources [14], with Facebook and other social media being commonly cited resources for new and transitioning mothers [15,16]. In addition to social media outlets, pregnancy-specific mobile apps have an important role in the self-health promotion of women and infants and are often consulted to search for signs or risks of illness [15]. Such apps may also promote well-being by reducing burden and feelings of isolation and improving health outcomes among new parents [16].

Despite the benefits of social media and pregnancy-specific apps, these sources of health information may have negative effects on new and transitioning mothers. For example, mothers who spend considerable time on Facebook after giving birth may experience increased stress, feelings of isolation, and depressive symptoms related to seeking external validation about their parenting practices [16,17]. Additionally, time spent consulting mothering websites was negatively correlated with mothers’ intentions to breastfeed [18,19]. Pregnancy-related mobile apps also present challenges to new mothers as these apps present wide variations in the trustworthiness of data and the privacy features [20]. For example, despite the increase in the number of pregnancy-related mobile apps designed to convey information about fetal movement, few apps provide explicit links between or clear instructions on how to interpret and respond to decreased fetal movement, the potential and likelihood of a stillbirth, or other adverse outcomes [21].

Further research is needed to explore parents’ use of digital technologies, why parents are going online, what they are viewing, and how it impacts their transition to parenting. Additionally, with issues concerning the access of reliable health information available from diverse digital sources, it is important to investigate parents’ use of health information technology to inform safe practices and recommendations for trustworthy and reliable health information access [22]. The purpose of this study was to enhance our understanding of the ways digital technologies contribute to the experience of the transition to parenting, particularly the role these technologies play in organizing and structuring emerging pregnancy and early parenting practices.

## Methods

A qualitative descriptive study was conducted to understand new parents’ experiences with and uses of digital technology during 4 stages—prenatal, pregnancy, labor, and postpartum—of their transition to becoming a new parent [23].

### Recruitment

This study took place during 2019 in an urban setting in Southwestern Ontario, Canada. As of 2016, the median after-tax income of couple families with children in this region of Ontario was CAD $84,608, whereas the median after-tax income of lone-parent families was CAD $45,952 [24]. A currency exchange rate of CAD $1=US $0.78 is applicable. A purposive convenience sampling strategy was implemented to recruit participants who had recently transitioned to becoming a parent within the previous 24 months [25]. Recruitment flyers were posted in locations where new parents were believed to frequent, such as local public health units, daycare centers, family health clinics, and early year play centers. Digital flyers and advertisements were also purchased online through buy-and-sell websites and social media platforms such as Facebook. Interested individuals were eligible to participate if they met the following inclusion criteria: (1) identified as a new parent who had recently undergone the transition to parenting within the last 24 months, (2) were between 16 and 35 years old, and (3) were fluent English speaking. The participant age limit was set to 35 years old as older parents, in particular mothers, constitute a generationally different cohort in terms of their technology use, health care needs, and health risks. All participants provided written informed consent prior to participating in this study and were provided with a CAD $15 honorarium immediately after signing the consent forms before engaging in data collection.

### Data Collection and Analysis

Focus groups and individual interviews were conducted by members of the research team in locations agreed upon between participants and researchers, including public libraries, a shelter, and a children’s center. The nature of inquiry within the focus groups was related to participants' use of digital technologies (ie, social media, use of pregnancy or parenting apps, participation in online pregnancy or parenting support groups). If a focus group participant introduced a topic that required additional time or consideration to discuss, an individual follow-up interview was offered. For example, such topics included in vitro fertilization and egg donation. A demographic questionnaire was given to each participant at the outset of the focus group to elicit descriptive characteristics of the participants. Data were digitally recorded and transcribed verbatim with thematic data analysis. Field notes by researchers were also employed to document relevant data not able to be captured by the digital recording, such as nonverbal communication.

Recruitment of focus group participants continued until data saturation was met [26,27]. An iterative, thematic analysis approach was used to coconstruct study findings [28,29]. All members of the research team analyzed each interview transcript, then together cross-compared insights and negotiated emerging themes through face-to-face dialogue during subsequent team meetings. Codes were tracked in a tabular matrix using exemplar quotes from interview transcripts to demonstrate the meaning of the code. Data saturation was achieved once no new themes, patterns, nor codes were identified and when categories being analyzed became repetitive in nature with no new information being generated through additional focus group discussions [28]. 

Members of the research team included in the analysis process were academics with a diverse range of academic, professional, and personal life experiences as they relate to the transition to parenting. All but one member of the research team were parents, with children between them ranging in age from 7 to 30 years of age at the time the study was undertaken. Team members had varying levels of personal engagement with digital technologies to support parental decision making, and these personal experiences were utilized at times to delve deeper into a particular quote or theme that was emerging. Through dialogue between team members, we engaged in interrelational reflexivity wherein we questioned how our own positional power and social locations shaped our prior assumptions and how our knowing broadened or shifted through interaction with the data collected and emerging themes [30].

## Results

### Participant Characteristics

A total of 10 focus groups and 3 follow-up individual interviews were completed with 26 participants. There were 2 to 4 participants per focus group. While recruitment efforts targeted parents of all genders and sexual orientations, all participants identified as heterosexual women. Participants ranged in age from 17 to 35 years old, with 8 being 20 years old or younger, 4 being between 21 and 29 years old, and 10 being between 30 and 35 years old. There was a range of formal educational attainment, with 7 in the process of completing secondary school, 1 who had completed high school, 1 who had completed community college or apprenticeship, 10 who had completed a university undergraduate degree, and 2 who had completed a graduate degree. Employment status was reported by 20 participants, of which 9 participants were unemployed, 3 were employed part time, and 7 were employed full time. Participants’ household income also varied, as 4 reported a yearly household income of less than $20,000, 3 reported between $20,000 and $50,000, 4 reported between $50,000 and $99,999, and 5 reported household income over $100,000 per year. Half of the participants (13/26, 50%) were married, 7 were single and had never been married, and 1 was separated from her partner. The majority of participants (18/26, 69%) identified as Caucasian, and 3 participants identified as racialized.

Participants were invited to discuss the types of digital technology they used in their day-to-day lives in the context of their transition to parenting. Our analyses identified participants’ insatiable need to obtain health information, often through the use of online apps, to support reproduction, to inform their decision making, to validate their parental care practices, and as a means to simply cope with the increasing demands of new parenting. Participants reported prolific use of digital technologies to direct fertility (eg, ovulation timing), for information seeking regarding development of their fetus, to prepare for labor and delivery, and in searching for a sense of community during postpartum. See Table 1 for details regarding technologies used at each stage of the transition to parenting and the reasons participants used them.

**Table 1:** The types and reported use of technology during the transition to parenting.

Type and reported use		Preconception	Prenatal	Labor and birth	Postpartum
**Type of tech use**					
	Devices	Smartphone	Smartphone, tablet, Doppler	Smartphone	Smartphone, breast pump, baby swing, TV, baby monitor, angel care monitor, scent diffuser
	Online sites	Texting, Google	Texting, streaming services (eg, Netflix, YouTube), search engines (eg, Internet Explorer, Google), online registries (eg, BORN Better Outcomes Registry Network), social media (Facebook, Snapchat, Instagram)	Texting, social media (Snapchat)	Texting, The Milk Meg, Google, Motherisk, Pinterest, BORN (Better Outcomes Registry Network), Dr. Jack Newman - International Breastfeeding Centre (IBC)
	Apps	Period tracker, fertility tracker, Ovia	Bump, Omama, What to Expect, Baby Centre, 3D ultrasound	Pampers	Facebook groups, FaceTime, White Noise, YouTube, Baby Tracker, Safety First, Baby, Pampers, Let Go (buy and sell app), O Mama
Reason for use		Track menstrual cycle, access information regarding symptoms	Monitoring, documenting, learning about pregnancy, pregnancy announcement, distraction (entertainment when not feeling well)	What to bring to the hospital/what to expect, games to help with labor pains, entertainment, surveys	Help baby sleep (white noise), tracking baby habits (respiratory function), birth announcement, connect to resources for childcare, personal information and social support

Interestingly, participants expressed both the need for these technologies to assist them in the day-to-day demands of preparing for and undertaking parenting, yet simultaneously expressed concerns about their personal patterns of use and the potential negative impacts their use could have on infant development and attachment. The following sections explore how participants navigated these tensions.

### “Is This Normal; Is This Happening to You?!”

Participants in this study frequently described using their digital technologies to determine if their preconception, pregnancy, and postpartum experiences were “normal” relative to others. For example, participants regularly used online search engines to access information when feeling anxious:

I definitely Googled a lot of stuff. Like, we had been trying for two years and so, I was constantly, “is this supposed to happen?” “is this normal?” “is it supposed to look like this?”

Transcript 1

Others would use their technologies to seek validation from friends:

I would text my friends that have had babies to say, “is this normal?”

Transcript 1

Some participants who had been previously pregnant expressed that they avoided search engines because they assumed their current experiences were normal in relation to their own positive past pregnancy(ies):

…then my third [pregnancy], I was just too busy to Google anything, I just assumed everything was normal.

Transcript 10

In contrast, participants with prior negative pregnancy experiences frequently consulted resources and reported using search engines for information as a coping mechanism to manage stress and anxiety:

…everything went really well while I was pregnant and then… I had a miscarriage and that was really devastating and hard. So, I feel like at the beginning [of my second pregnancy], I was Googling a lot because I was like, “oh my God, what about this symptom?! The last time I felt that pain, this happened.” So, with [my second pregnancy], I was more paranoid.

Transcript 11

Despite the benefits that search engines potentially offered, some participants acknowledged that the information found through Google or other search engines could lead to false assumptions of normalcy that may endanger their and their fetus’ health. One participant described a situation where her husband assumed everything was normal because of her active Google use, while she used the information gleaned from the search engine to conclude that there was a potential abnormal issue developing:

I remember reading a lot about if you don’t feel your baby move, you should do this… I didn’t feel my son move one morning, and I was like, “I’m going to the hospital,” and my husband’s like, “you’re on Google, you’re fine.” But I wasn’t fine, and then I had him an hour later… I obviously found that [being on Google] helped me.

Transcript 11

In addition to using search engines to determine if their experiences were normal, participants in this study also used their digital technologies to facilitate communication with their health care providers to determine if their experiences were normal. For example, as one participant described:

I wasn’t sure what was going on and what would be necessary for the physicians to know, and I’m not really good with explaining things when I’m nervous or upset or have some anxiety about something. So, I took a picture of the spit up [with my phone] and took it to the hospital with me so they knew what I was talking about.

Transcript 2

Other participants described using their digital technologies immediately following medical procedures to interpret if the information shared with them by their health care professionals meant that their experiences were normal. For example, one mother described how she verified the normalcy of her daughter’s coloration immediately following birth:

My daughter was really red when she was born. Her skin was so red and purple, and I’m like, “is that normal?!” They were telling me that it’s just because she was just born and probably because she was born so quickly, and then I was just looking that up, “why is she so red?” But I just read that it’s pretty much normal for some babies to be really red and purple when they’re born.

Transcript 3

In a similar sentiment, other participants in this study described how social messaging outlets were a place to share their own expertise to help other mothers when they would ask questions about their experience:

I was on boards especially when I was going through the grief stuff... I would share my story about I’m diagnosed with Turner Syndrome and I’ve gone through all of this, and I had some girls message me that I don’t know saying “can you tell me about it? What do I have to look for in store for what my child?”

Transcript 8

Finally, participants in this study reported that while their use of digital technology increased, “I think I use technology more now that I have kids…” [Transcript 10], extending most often toward answering the question “Is this normal,” their partners’ behaviors often remained unchanged: “He uses social media in different ways. He connects with online gaming and those kinds of things, but not so much for parenting.” They also described how apps tailored to fathers were hypermasculinized to convey the size of the growing fetus:

It was like a daddy app, so it was like relating [the fetus] to a size of a beer or something like that. It was totally like dad style ... And then I think he would come to my pregnancy app to look at it if he wanted it to be a bit more serious.

Transcript 12

### “Am I Having a Heart Attack; What is This?”

Participants described how digital technologies made it easier and more convenient to communicate with others about their pregnancy and enabled them to find responses to their questions and health information needs more rapidly and during all hours of the day than other communication channels. For example, digital technologies facilitated visual communications between family members and enabled geographically distant family members to interact with the participants’ children:

[We use] Facetime a lot… Especially like my mom, they go to Florida for the winter, so to keep in contact with them, just we’ll Facetime once every couple of days so they can see him [infant].

Transcript 11

Digital technologies also offered a convenient means for participants to share health information with health care professionals:

In the beginning, during breastfeeding and when I got in touch with the lactation consultant… we communicated via text message, and I would ask her like, and I know it sounds weird, but she would be like “send me a picture of him feeding so I can see.” So, I would send her a picture and she would tell me like “adjust his head” or “put your hand this way” or whatever.

Transcript 11

Social media websites were often highlighted for the quick responses that participants received from other users when in search of health information about their pregnancy-related experiences:

I did a lot of research online about egg donation and in vitro and found out through Facebook through a friend of mine… about her surrogacy journey, so I ended up contacting her online to find out more about the agency that she worked through.

Transcript 8

Additionally, the convenience of digital technologies was described as an important element that helped participants address their stress caused by uncertainty around their own health and well-being:

I did Google once at like 3 in the morning. I had, like it was heartburn, but that was like – I have never had it before and I was “am having a heart attack?! What is this? This is way more intense than I thought heartburn would be.” So, I did Google and read some stories of other people and just “okay, this is pretty intense.”

Transcript 6

Not all participants found social media to play a positive role in their parenting journey and did not post online out of fear of judgement or concerns about being perceived as not measuring up to the social norms expected of “good mothers.” As one participant shared:

My house is a mess in the background, and I’m not posting that. Or why are you making that smile or it’s blurry? He’s [baby] goofy and not wanting to take a picture.

Transcript 4

For another participant, the shelter where she was staying forbid the use of digital technologies:

I had no phone when I got there, like I was ready to leave. I had no contact with anybody, and I kind of built myself up but, like I said, they had no internet and they refused to get internet because their thing was – it’s like a maternity home where like, you know, like kids are there. They’re like “you shouldn’t be on your phones while you’re playing with your kids,” and technically that’s what you’re supposed to be doing all the time.

Transcript 5

### “Anyone Can Put Anything on Wikipedia”: Managing the Negative Impacts of Digital Information

While each participant noted a positive aspect of digital technology usage during the transition to parenting (eg, staying connected to family, obtaining health information, receiving validation from peers), participants also expressed apprehension about the credibility of the digital sources of health information, how to best use these sources, and whether or how to act on information they retrieved. There was some concern about trusting health information on certain websites due to the lack of transparency of the authors’ credentials and expertise in the health care field. One participant compared the trustworthiness of health information found on crowd-sourced websites to that found on health-specific websites:

Anyone can put anything on Wikipedia. They can change all the information, like you can go on and change it yourself. So, it’s like the health websites is usually actually there’s a nurse answering your question.

Transcript 3

Another participant describes how she lost trust in non-health–specific websites and now only trusts websites published by health care organizations:

I did trust it at one point and then people were telling me that people can go in and change the information. So, once I heard that, like multiple times, through growing up, I do not really trust it. Some information, like the health unit website and what not, that is the type of information that I would check.

Transcript 7

Participants described how the amount of health information available through digital technologies was often overwhelming, which created uncertainty regarding how to interpret or act on the information they found. One participant described how these feelings during postpartum were alleviated by support to do something with the information she found:

Postpartum is just such an intense time that I feel like it—like there’s so much information and different information. The support isn’t really there.

Transcript 12

Due to the amount of health information sources, participants described the need to critically appraise websites to ensure they were obtaining credible information:

You have to look at who’s sponsoring the article, right? Because you can literally find any information that you want to hear or see. So, you have to really know how to dissect even the science-based studies.

Transcript 12

Similarly, participants noted the importance of questioning the credibility of health information they received through social media groups or message boards as some information may inappropriately exacerbate their anxieties:

I had a massive bleed at the beginning of my pregnancy, and I thought I was miscarrying. So, of course, I was writing on this and everyone’s like, “oh you’re probably miscarrying. You should check it out.” But then it turned out it was fine… I think it can be like anxiety and comforting at the same time because I feel like any symptom can be put in, it can be either completely abnormal or completely normal.

Transcript 11

Some participants described strategies to verify the accuracy of health information they found through digital technologies as a way to ensure they were acting on credible information. For example, checking multiple sites for answers to a question was a common approach to verify health information:

If I Google something, I never just go for the first answer. I always have to check out 4 or 5 different websites and, you know, if they all say the same thing then that tells me I think that’s a good thing to follow.

Transcript 2

Participants did note that consulting some nontechnological resources—such as health care providers, books, pamphlets, prenatal classes, their public health nurse, other moms, family, and friends—was a method to verify information obtained online. Of all nontechnological resources, participants’ physicians were viewed as their most trustworthy source for health information and as a credible third-party to verify the accuracy of information found online:

So, I thought this Doppler I got like if I could hear the heartbeat that I would feel better. So, I didn’t know how to do it, so I had to YouTube it… If I had to, I would go to the doctor, because that’s essentially—I would never leave it up to my Googling or my experience with a Doppler to determine if it was valid or not, or if I was doing it right. If I was in doubt, I would go see a professional. I would never take Google’s word or YouTube’s over mine, but it’s helpful.

Transcript 11

## Discussion

This investigation of digital technology use in the transition to parenting singularly highlights mothers’ use of digital technologies across preconception, prenatal, labor, and birth and during postnatal stages. For mother participants in this study, substantial effort was given to understand “Is this normal?” which is consistent with existing literature on mothering [31,32]. Experts argue that the ongoing search for information is generated, in part, by societal norms that prescribe women to parent with the pressure to be perfect, contributing to the toll on mothers’ well-being [31,32]. Participants’ use of digital technologies in the transition to parenting was accompanied by feelings of constant negotiation, of trade-offs between the relative ease and instantaneous access to answer the question “Is this normal?” with potential downsides, such as the likelihood that the information could be inaccurate or potential judgement. Our study contributes to this literature by demonstrating the ways that participants extended their search for answers beyond the traditional sources. Participants in this study turned to digital technologies, online platforms, apps, forums, and streaming services to answer this relentless question, with most participants accessing information on their cell phones for convenience and because of their ubiquitous presence.

Consistent with other research [33,34], mothers in this study demonstrated an unrelenting drive to obtain health information online: to direct fertility (eg, ovulation timing), for information seeking regarding development of their fetus, to prepare for labor and delivery, and to generate a sense of community during postpartum. In effect, they “Googled” everything. This was especially true for first-time mothers and mothers with a history of negative pregnancy experiences. Across the stages of the transition to parenting, apps, videos, online shopping, and forums were important sources of tangible and intangible information, resources, and services accessible regardless of place and time. In considering increased patient empowerment with the possibilities for greater self-management of prenatal and postpartum care, digital technologies have the potential to enhance efficiency of care and contribute to the revolution in perinatal care [35].

However, our findings suggest that opportunities for enhanced self-management and empowerment may not be equitably obtainable. Consistent with participants in our study, many people feel compelled to search for health information online especially when they experience difficulties in accessing health care services [36]. Despite concerns about the overwhelming amount and questionable trustworthiness of online information, mothers in our study favored the immediacy and convenience of digital information (eg, internet, apps) expressly when health care services were less accessible (ie, middle of the night). In fact, mothers minimized their concerns regarding misinformation and privacy violations in search of validation or direction when the need for information in response to a health concern was perceived as pressing. Our findings add to the research by Amante et al [37], who reported that primary care patients used online information to determine their need for health care services or for self-health management (eg, alter or cease prescribed treatments). Different than the findings reported by El Sherif et al [38], mothers in this study used their digital technologies to communicate with health care providers and online parenting peers (ie, send images of health concerns) to determine if their experiences were normal and the need for additional intervention. In some instances, the online communication mitigated the need for in-person health service consultation (ie, online breastfeeding consult). Additional research is needed to fully understand the antecedents (eg, digital health literacy skills) and consequences (eg, health outcomes, health service inequities) of individuals’ use of online health information especially within the context of patient-centered care practices and health service utilization patterns.

As well, some participants in this study conveyed a sensible skepticism regarding the credibility of digital sources of health information. The strategies employed by some of the mothers in this study (eg, assessing the website sponsor, access known government or health organization sites, assess consistency of information across multiple sites or sources) to confirm the trustworthiness of online information resources align with nationally advocated guidelines [39,40]. Yet the online information-seeking challenges for parents remain significant. Despite their awareness of online misinformation, mothers were challenged in their ability to discriminate accurate from false health information. Consider how antivaccination propaganda is amplified online, with life-threatening consequences to infants, children, and the wider community. Researchers have found that information from online sites has influenced decisions whether to vaccinate [41,42], and parents with the greatest need for knowledge about vaccination are seen as most vulnerable to false online health information [43]. Similarly, Ashfield et al [42] reported that parents found online information posted by antivaccination groups as very scientific in appearance (scientific language and academic formatting), making it increasingly difficult to determine credible from misleading information.

Beyond the ability to appraise online health information, we recognized the need for enhanced digital health literacy skills among the mothers in our study. Defined as the ability to seek, find, understand, and appraise health information from electronic resources and apply the knowledge gained to address or resolve health issues, digital health literacy skills involve the mastery of multiple literacies: traditional literacy, health literacy, information literacy, scientific literacy, media literacy, and computer literacy [44]. Like mothers in this study, Lee and Moon [13] found that online health apps were an important source of information for pregnant women. In their study exploring the use of 47 mobile apps for pregnancy, birth, and childcare, they determined that apps have become an important information source for pregnant women, more frequently used when searching for information concerning signs of risk and disease. Concernedly, of the criteria used to evaluate the usability of the apps (eg, information clarity and protection), “the information source” had the lowest score. They concluded that the quick provision of health information was desired and seen as a motivator, but often credible professional information was sorely lacking [13].

Our findings demonstrate that mothers’ use of digital health technologies have the potential to move parents beyond self-care practices and into the scope of clinical practice. Furthermore, the “health care work” of mothers has the potential for health-enhancing outcomes, but also dire consequences. Parents’ use of digital health technologies without advanced clinical knowledge and skill highlights the risk of adverse events as in the “near miss” described by one mother who noted a lack of fetal movement but was reassured by her partner that the information she found online discounted the need for health care intervention. A false sense of confidence or reassurance when accessing potentially inaccurate health information disseminated online or an inability to appraise and apply the health information to their specific situation may lead to delayed access to health services or increase the need for costly health care services when an emergency arises [38,45]. Additional research is needed to better understand the implications for the health outcomes and health service utilization patterns among individuals in the transition to parenting within the digital health context.

### Limitations

While this study provides valuable insight into how new parents perceive the use of digital technologies as they transition to parenthood, it is not without its limitations. Importantly, while this study aimed to recruit all parents, participants reflect a single type of parenting perspectives: those of heterosexual women. Although this sample presents an in-depth description regarding mothers’ use of digital technologies during the transition to parenting, further research is needed to more fully understand this health information–seeking process and its unintended consequences for gendered health work. Future studies that target different types of parents, such as heterosexual men, LGBT men and women, and gender-fluid men and women and parents of different cultures, race, and language other than English may help further elucidate the nature of digital technology use while transitioning to becoming a parent.

### Implications for Education, Practice, and Research

This research has implications for enhanced development of parents’ digital health literacy skills; parents require the skills to be able to identify misinformation resulting from their online information seeking. Developers of prenatal education programs should consider the importance of digital health literacy skill enhancement and to generate online information to accommodate a range of parental digital health literacy skills. This work also has implications regarding health education and clinical assessment by health care providers; providing credible online resources constitutes an important health education strategy for information-seeking parents.

Further research is needed to understand the nuanced practice and policy impact of mothers’ digital technology use and access to health care services. Further studies that create targeted advertisements to different types of parents, such as cis heterosexual men, 2LGBTIA+ families, folks who are gender nonbinary, and parents of different cultures, race, and language other than English may help further elucidate the gendered nature of digital technology use while transitioning to becoming a parent. For example, further research should seek to understand how men and fathers use digital technologies during their transition to parenting. Doing so may illuminate possible avenues to meaningfully engage men in the health information–seeking process and promote a sharing of health work within a parent dyad. Additionally, further research should critically examine how constant access to information affects mothers’ perceived need to self-monitor as they transition to parenting. Such research may provide a deeper understanding of the unintended health consequences of constant surveillance on new parents. Together these understandings may provide health care providers and decision makers with information required to appropriately regulate how different health information can be shared with specific populations.

### Conclusion

This study provided a descriptive analysis of how new mothers utilize different forms of digital technology as they transition to becoming a new parent. A range of technologies was used at each stage of the transition, with smartphones being ubiquitous across all stages. Digital technologies provided participants with convenient means to access health information from a range of sources such as websites, online support groups, and health care professionals. While digital technologies made health information access more convenient, participants were apprehensive about the credibility and trustworthiness of the information they retrieved due to limited transparency in the authors’ expertise and credentials.
